# An Overview of the Impact of Bacterial Infections and the Associated Mortality Predictors in Patients with COVID-19 Admitted to a Tertiary Center from Eastern Europe

**DOI:** 10.3390/antibiotics12010144

**Published:** 2023-01-11

**Authors:** Amalia-Stefana Timpau, Radu-Stefan Miftode, Irina-Iuliana Costache, Antoniu Octavian Petris, Ionela-Larisa Miftode, Liliana Gheorghe, Razvan Timpau, Ioana Diandra Miftode, Cristian Sorin Prepeliuc, Ioana Coman, Dana-Teodora Anton-Paduraru, Cristina Tuchilus, Egidia Gabriela Miftode

**Affiliations:** 1 Department of Infectious Diseases (Internal Medicine II), Faculty of Medicine, University of Medicine and Pharmacy “Gr. T. Popa”, 700115 Iasi, Romania; 2 Department of Internal Medicine I (Cardiology), Faculty of Medicine, University of Medicine and Pharmacy “Gr. T. Popa”, 700115 Iasi, Romania; 3 Department of Radiology, Faculty of Medicine, University of Medicine and Pharmacy “Gr. T. Popa”, 700115 Iasi, Romania; 4 Radiology Department, St. Spiridon Clinical Hospital, 700115 Iasi, Romania; 5 St. Parascheva Clinical Hospital of Infectious Diseases, 700116 Iasi, Romania; 6 Department of Mother and Child Medicine, Faculty of Medicine, University of Medicine and Pharmacy “Gr. T. Popa”, 700115 Iasi, Romania; 7 Department of Preventive Medicine and Interdisciplinarity (Microbiology), Faculty of Medicine, University of Medicine and Pharmacy “Gr. T. Popa”, 700115 Iasi, Romania

**Keywords:** COVID-19, bacterial infections, antibiotherapy, mortality predictors

## Abstract

1. Background: Literature data on bacterial infections and their impact on the mortality rates of COVID-19 patients from Romania are scarce, while worldwide reports are contrasting. 2. Materials and Methods: We conducted a unicentric retrospective observational study that included 280 patients with SARS-CoV-2 infection, on whom we performed various microbiological determinations. Based on the administration or not of the antibiotic treatment, we divided the patients into two groups. First, we sought to investigate the rates and predictors of bacterial infections, the causative microbial strains, and the prescribed antibiotic treatment. Secondly, the study aimed to identify the risk factors associated with in-hospital death and evaluate the biomarkers’ performance for predicting short-term mortality. 3. Results: Bacterial co-infections or secondary infections were confirmed in 23 (8.2%) patients. *Acinetobacter baumannii* was the pathogen responsible for most of the confirmed bacterial infections. Almost three quarters of the patients (72.8%) received empiric antibiotic therapy. Multivariate logistic regression has shown leukocytosis and intensive care unit admission as risk factors for bacterial infections and C-reactive protein, together with the length of hospital stay, as mortality predictors. The ROC curves revealed an acceptable performance for the erythrocyte sedimentation rate (AUC: 0.781), and C-reactive protein (AUC: 0.797), but a poor performance for fibrinogen (AUC: 0.664) in predicting fatal events. 4. Conclusions: This study highlighted the somewhat paradoxical association of a low rate of confirmed infections with a high rate of empiric antibiotic therapy. A thorough assessment of the risk factors for bacterial infections, in addition to the acknowledgment of various mortality predictors, is crucial for identifying high-risk patients, thus allowing a timely therapeutic intervention, with a direct impact on improving patients’ prognosis.

## 1. Introduction

Infection with severe acute respiratory syndrome coronavirus 2 (SARS-CoV-2) caused over 6.5 million deaths by November 2022, having a major impact on medical, educational and socio-economic systems [1]. One of the public health issues exacerbated by the ongoing pandemic is the bacterial antimicrobial resistance (AMR) phenomenon, with its precise extent being currently under extensive investigation [2]. The World Health Organization (WHO) reported that half of the antibiotic regimens have been incorrectly prescribed worldwide, with the primary consequence being the worrisome growing trend of AMR [3]. High rates of antibiotic use were reported in COVID-19 patients, both in-hospital and in outpatient care facilities, particularly during the early phases of the pandemic [4,5]. The lack of adequate diagnosis tools for ruling out bacterial co-infections at admission, doubled by the considerable risk of bacterial secondary infections, represents the leading causes of antibiotic misuse [6,7]. The widespread antibiotic use without medical advice, self-medication with antibiotics, their use in inappropriate dosages, or improper therapy length have reportedly increased since the beginning of the pandemic [8]. Moreover, the deficiency of certain antibiotic treatments in several geographic areas and natural bacterial resistance patterns further increase the burden of AMR [9].

Significant literature data have documented the prevalence of bacterial infections, antibiotic prescriptions, and their mutual interdependence during the ongoing pandemic [6,10]. A study conducted by Rawson et al. reported antibiotic use in almost three quarters of the analyzed COVID-19 patients in the early pandemic period, with only 8% of cases having culture-proven bacterial co-infection or secondary infection [10]. The current paradigm is aiming to identify, in a timely manner, the risk factors for bacterial co-infections. A study led by Morenno-Torres et al. claims that advanced age, neurological impairment, immunosuppression, and ICU admission are significant predictors for co-infections [11]. Current reports indicate *Pseudomonas aeruginosa* and *Acinetobacter baumannii* as the microbial strains that often cause nosocomial infections and are also the main agents of bacterial infections in COVID-19 patients [6,12,13].

The SARS-CoV-1 epidemic has left its mark on the pattern of causative agents for secondary infections, by inducing an increase in the prevalence of multi-resistant strain infections [14]. In the framework of substantial antibiotic overuse in COVID-19 patients, the long-term impact of the SARS-CoV-2 pandemic on AMR is essential and represents an important subject for further research [14,15]. In Romania, the AMR level is increasingly worrying, far above the figures reported in Western Europe. The contributors to this phenomenon are antibiotic abuse and the widespread prescription of broad-spectrum agents in hospital settings [16]. In this regard, the implementation of antibiotic stewardship protocols in COVID-19 patients is of paramount importance in overcoming the AMR-associated burden, especially since the discovery of new agents for the treatment of multi-resistant infections is not highly promising [17].

Considering all these aspects and the increasing focus on judicious antibiotic prescription, in this study, we aimed to highlight several particular aspects associated with antibiotic administration in COVID-19 patients, as well as to assess their outcome by identifying significant mortality predictors. The study’s main aim was to investigate the incidence of bacterial infections in patients with COVID-19 and their associated risk factors. Secondly, we also emphasized the identification of mortality predictors and the evaluation of biomarkers’ abilities in predicting a poor outcome. In this context, the study was conducted in the north east region of Romania, an “endemic” area for antibiotic resistance, where several microbiological, epidemiological, and socio-economic factors play a major role in defining the AMR phenomenon [18,19].

## 2. Results

### 2.1. Baseline Characteristics and Comorbidities

The average age of the patients was 60.4 years ± 15.2, with a similar gender distribution, as 49% of the patients were male (Table 1). In total, a moderate form of the disease was diagnosed in 52% of cases and a severe form in 35%, while a critical form was found in only 13% of cases, without statistically significant differences between the two cohorts. The median length of stay of the patients who received antibiotics was significantly longer compared to the control group (11 days (IQR 10–11) vs. 9 days IQR 6–9) (*p* = 0.032)). Associated pathologies were documented in 202 patients (73%). The most prevalent comorbidities were cardiovascular, which were identified in 135 patients (48.2%), followed by obesity and diabetes, in 68 (24%) and 62 cases (22.1%), respectively, with significant differences between the two groups regarding the prevalence of obesity (*p* < 0.01) and cardiovascular comorbidities (*p* < 0.01). At the time of hospital admission, 125 (44.6%) patients demonstrated peripheral oxygen saturation of ≤93% in room air at sea level and 119 (42%) had an axillary temperature above 37.5 degrees Celsius, with no significant differences between the two groups.

### 2.2. Laboratory and Imagistic Findings

Significantly higher levels of inflammatory biomarkers such as CRP (*p* < 0.01), fibrinogen (*p* = 0.01), and procalcitonin (*p* = 0.005) were detected in patients treated with antibiotics (Table 2). Concerning D-dimers and ferritin levels, we found no significant differences between the two groups. Chest CT findings, comprising both ground-glass opacities and pulmonary consolidations, were significantly more prevalent in the antibiotic-treated group. 

### 2.3. Microbiological Profile of COVID-19 Patients 

**Figure 1:**
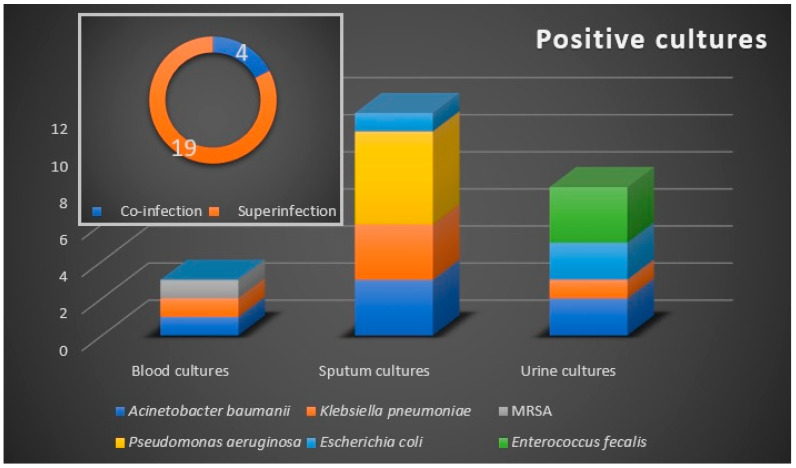
Bacteriological profile with the distribution of co-infections and superinfections. Microbiological samples are blood, sputum, and urine. Etiologic agents are marked by color and they are allocated according to the confirmed microbiological sample.

The microbiological samples were positive in 23 (8.2%) patients (Figure 1), with a prevalence of 4 (1.4%) cases of bacterial co-infection, and 19 (6.8%) cases of bacterial secondary infection. The co-infection cases were represented by urinary tract infections, while respiratory tract bacterial secondary infections were confirmed in 12 (4.28%) patients. Urine cultures and hemocultures were positive in eight (2.85%), and three (1.07%) patients, respectively.

The microbiologically confirmed bacterial infections were exclusively recorded in the antibiotic-treated group. Six bacterial strains were identified as etiologic agents, with the Gram-negative bacilli *A. baumannii, P. aeruginosa,* and *Klebsiella pneumonia* being the most commonly isolated pathogens. We noted an increased rate of bacterial infections in critically ill patients, with 52% of cases demonstrating secondary infections that required ICU admission, of whom 33.3% have died. 

Of the 24 microbiologically confirmed secondary bacterial infections, 6 (25%) were with multidrug-resistant (MDR) Gram-negative bacilli strains. Of these, three infections were respiratory and three were urinary infections. No extensively drug-resistant or pandrug-resistant strains were identified. Two isolates were extended-spectrum cephalosporin-resistant, while one isolate was resistant to carbapenems.

### 2.4. Therapy and Evolution

**Figure 2:**
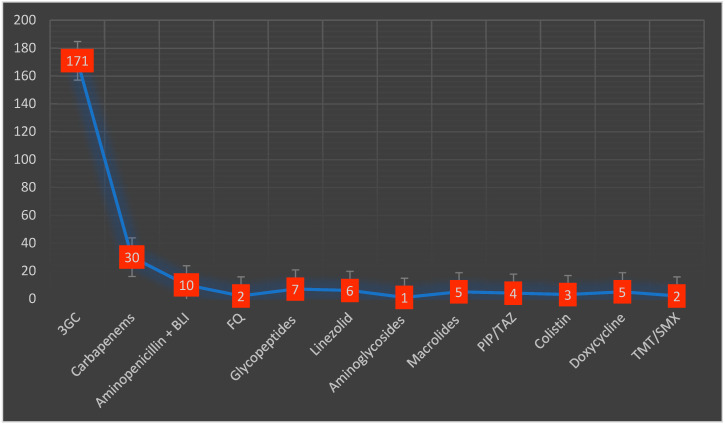
Types and distribution of antibiotics administered during the hospital stay. 3GC—third-generation cephalosporins, BLI—beta-lactamase inhibitors, FQ—fluoroquinolones, PIP/TAZ—piperacillin/tazobactam, TMT/SMX—trimethoprim/sulfamethoxazole.

Almost half of the included patients (42%) received antibiotic therapy before admission. Macrolides were the most used antibiotic class in 53 cases (18.9%), followed by second-generation cephalosporins in 34 patients (12%). During their hospital stay, empiric antibiotic treatment was administered in 204 (72.8%) patients. Third-generation cephalosporins were the most frequently prescribed in 171 (73.5%) patients, followed by carbapenems in 30 (13.2%) patients (Figure 2). The average duration of the antibiotic treatment was 8.5 days (3.2). Adjustment of the antibiotic treatment was necessary in 59 cases (25%). Inappropriate empiric treatment required adjustments mostly after the administration of third-generation cephalosporins, usually being replaced by carbapenems. The average number of antibiotics prescribed per patient was 1.4.

Antiviral treatment was significantly more commonly prescribed in the patients who received antibiotics, compared to the control group (*p* = 0.042) (Table 3). Monoclonal antibodies, such as anakinra and tocilizumab, were administered in 40 (14.2%), and 48 (17.1%) patients, respectively. There were no significant differences between the two groups in terms of monoclonal antibodies or glucocorticoid use.

Concerning the outcome, patients who received antibiotics had a significantly higher mortality rate (*p* = 0.01), compared to the control group (Table 4). 

### 2.5. Predictors of the Need for Antibiotic Treatment in COVID-19 Patients

First, we assessed the correlation between the antibiotic treatment and some relevant clinical and biological parameters (Table 5). We observed that the need for antibiotic treatment was significantly correlated not only with hypoxia at admission (R = −0.165, *p* = 0.006), but also with the coexistence of chronic comorbidities, such as diabetes mellitus (R = 0.170, *p* = 0.004), or cardiovascular pathologies (R = 0.340, *p* < 0.001). It is worth mentioning that antibiotic treatment was the most significantly correlated with pulmonary consolidation on CT. Concerning the laboratory profiles, the presence of hyperglycemia, leukocytosis, and high inflammatory markers (e.g., CRP and fibrinogen) at admission was directly correlated with the need for antibiotic administration. Interestingly, increased D-dimers or impaired renal function did not significantly influence the need for antibiotic therapy. 

Subsequently, the parameters that were significantly correlated with antibiotic treatment were included in a multivariable logistic regression that provided a stepwise statistical model. We found that a model that comprises the pulmonary consolidation on CT and the presence of cardiovascular comorbidities would play a significant role in predicting the need for antibiotic therapy (R = 0.505; R^2^ = 0.255), by highlighting that the coexistence of these two findings may significantly increase the underlying infectious risk and the need for subsequent antibiotic treatment (Table 6).

### 2.6. Risk Factors for Bacterial Infection and Mortality

We conceived a multivariate logistic regression model to establish the risk factors for bacterial infection (Table 7). ICU admission and leukocytosis were identified as significant predictors and the preliminary Hosmer and Lemeshow test validated that the model adequately fit the data (chi-square = 6.563, *p* = 0.584).

CRP and length of hospital stay were the main risk factors associated with increased mortality, as confirmed by the multivariate logistic regression (Table 8).

### 2.7. Role of Biomarkers in the Assessment of Mortality Risk

**Figure 3:**
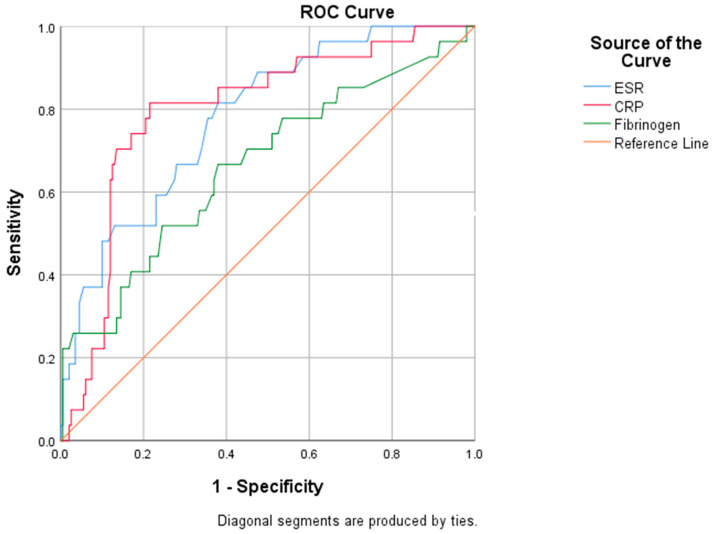
ROC analysis for specified biomarkers.

We evaluated the diagnostic performance of inflammatory biomarkers by performing an ROC analysis (Figure 3). 

The subsequent areas under the curve (AUC) revealed the adequate performance of ESR (AUC: 0.781) and CRP (AUC: 0.797) in predicting mortality risk, while fibrinogen (AUC: 0.664) exhibited an inferior predictive ability (Table 9).

We further extracted from the ROC curves several relevant cut-off values, such as the value that indicates the maximum sum between sensitivity and specificity (Youden’s index), or the threshold associated with increased mortality (Table 10). A reliable cut-off for CRP in identifying patients with a high mortality risk was established at 67.01 mg/L, with a sensitivity of 74.1% and a specificity of 53.5%. Concerning ESR, a value of 51.5 mm/h predicts the risk of mortality, characterized by a sensitivity of 74.1% and a specificity of 56%.

## 3. Discussion

There are scarce literature data available concerning the bacteriological profile of secondary infections and antibiotic use in COVID-19 patients admitted to Romanian hospitals. In the framework of this study, we found rather reduced rates of bacterial co-infections and secondary infections (of 1.4% and 6.8%, respectively) compared to a meta-analysis study conducted by Langford et al., where the reported figures were significantly higher (of 3.5% and 14.3%, respectively), but similar to the prevalence rates observed in other cohorts [6,11,20]. 

In our study, the high rates of pre-admission antibiotic therapy in 42% of the patients might have negatively influenced the diagnosis of bacterial infection. Prior antibiotic exposure was associated with late hospital presentation, a more frequent need for oxygen therapy, and a lower anti-COVID-19 vaccination rate [21]. In line with previous studies, we found that the most commonly isolated microbial strains were Gram-negative bacilli, such as *A. baumannii, P. aeruginosa,* and *K. pneumoniae* [13,22]. In COVID-19 patients, the intrinsic risk of superinfection is substantially higher than the risk of co-infection, an aspect highlighted not only by our study, but also by a growing body of literature data, thus confirming that the associated bacterial respiratory tract infections are nosocomial rather than community-acquired [6,23]. 

An increased risk of *A. baumannii* infections has been observed since the SARS-CoV-2 outbreak and the mortality associated with this etiologic agent is worrisome, given that it has been reported to be as high as 85.7% [24]. *P. aeruginosa* emerged as the main etiology for bacterial respiratory tract infections. A study that investigated the nasal microbiome of SARS-CoV-2-infected patients identified a larger number of microbial strains, including *P. aeruginosa*, in the nasal cavity, as compared to those without viral infection. This abundance and diversity of pathogens, which are frequently directly correlated with viral load, might play a role in the growing trend of secondary bacterial infections [25]. Assuming that infections in patients with COVID-19 are habitually caused by multidrug-resistant strains, an experimental study designed an antigen using epitopes of SARS-CoV-2, *A. baumannii,* and *P. aeruginosa*. The idea of a multi-target antigen that is able to trigger protective antibodies is attractive for facing multidrug-resistant pathogens, but additional studies are further needed to ascertain its safety and efficacy [26]. Romania ranks high amongst countries with a high antibiotic consumption per capita. Nationally, multidrug resistance remains high for *K. pneumoniae,* but has decreased for *Escherichia coli* [27]. Even if the characterization of the various antibiotic resistance patterns was not the objective of the present paper, we still mention that in a study carried out in the very same hospital, Miftode et al. reported a higher prevalence of Gram-negative bacilli-resistant strains that were involved in the etiology of urinary tract infections in patients from the north-east region of Romania [28,29]. Additionally, in the same geographical area, increasing rates of urinary infections, with *K. pneumoniae* producing extended-spectrum beta-lactamase, were reported [30]. These data are also confirmed by other studies that investigated the antibiotic resistance profiles of the Romanian population, thus outlining an increased incidence of urinary tract infections with MDR strains of *E. coli* and *K. pneumoniae* [31].

By using the indicators recommended by the WHO, we evaluated the average number of antibiotics taken by each patient and identified an optimal rate of 1.4, with the recommended limit being 1.6–1.8 [32]. Even though only a fraction of patients had a culture-confirmed bacterial infection, the majority of patients received empiric antibiotic treatment. The current national and international guidelines recommend the use of empiric antibiotic therapy in patients with mild or moderate forms of the disease only if the bacterial infection is clinically suspected or in the presence of biological or imaging indicative evidence. Conversely, for patients with severe forms of the disease, the guidelines recommend adapted antibiotic therapy to cover all the likely pathogens [33,34]. Several studies have investigated the impact of antimicrobial therapy on COVID-19 patients’ mortality rate, concluding that antibiotic use does not reduce the risk of a fatal outcome [35,36]. Furthermore, the use of certain antibiotic classes is per se associated with an increased mortality risk, especially in patients with cardiovascular comorbidities, with macrolides bearing the greatest burden in this respect [35,37]. In our study, cardiovascular comorbidities represented a significant predictor for the antibiotic treatment need of the patients, providing additional predictive value when included in a composite multivariable model. 

Patients admitted for COVID-19 receive antibiotics that are used in the management of community-acquired pneumonia, such as amoxicillin/clavulanate, macrolides, or ceftriaxone, as well as broad-spectrum antibiotics, despite the low rate of confirmed associated bacterial infection [38,39,40]. In line with previous data, in our study, we noted that the most frequent antibiotics used during a patient’s hospital stay were the broad-spectrum regimens, namely third-generation cephalosporins, and carbapenems [6]. We documented the use of macrolides as the most common class of antibiotics administered before admission. This aspect could be explained by the consistent research during the early pandemic concerning the effect of macrolides in patients with SARS-CoV-2 infection. However, some results indicate that macrolides are ineffective for viral pneumonia, and consequently have no benefit regarding the patients’ prognosis [41]. The decision to initiate antibiotic treatment in SARS-CoV-2-infected patients is challenging, mainly because the clinical features and CT aspect of viral pneumonia might be similar to the respiratory infections produced by *Streptococcus pneumoniae* or *K. pneumoniae* [42,43]. Secondly, procalcitonin, despite being a biomarker with a better predictive value compared to CRP or interleukin-6 in differentiating between bacterial and viral pneumonia, still has a rather low reported sensitivity and specificity, of 0.55 and 0.76, respectively [44]. Several studies suggest that procalcitonin is not a reliable indicator of bacterial infections in patients with COVID-19, but should be rather used as a surrogate marker for risk stratification [45,46]. Therefore, the rate of inappropriate use of antibiotics, especially broad-spectrum antibiotics, remains high in the context of using classic diagnostic tools [17]. Within this framework, the realistic priority in combating AMR is to de-escalate to a narrow-spectrum antibiotic once the microbial strain sensitivity is tested [47].

In this research, by performing a univariate analysis, we highlighted the significantly higher values of the inflammatory biomarkers, such as CRP, fibrinogen, and procalcitonin, in patients who received antibiotic treatment and their substantial prognostic value and their subsequent contribution in treatment decision-making was well-recognized [48]. The ROC curve revealed the acceptable performance of CRP (AUC: 0.797) and ESR (AUC: 0.781) in predicting the mortality risk, while fibrinogen exhibited a rather modest predictive value (AUC: 0.664). Strong evidence supports the hypothesis that high ESR levels are a significant negative prognostic factor in severe forms of the disease. Despite its low specificity, an elevated ESR might provide additional information on disease progression [49,50,51]. Regarding the widely used CRP, the present study confirmed the previous data reported by our team in which we found a similar performance of CRP in predicting the mortality risk [52]. For initial prognostic assessment, we identified that a CRP concentration of 67 mg/L is a reliable “high-risk” cut-off, a value similar to that reported by other studies [53]. An alternative CRP threshold of ≥40 mg/L at admission may be equally useful for clinicians in assessing disease severity and stratifying the mortality risk [54].

A dysregulated and impaired inflammatory response is a common finding amongst patients that require ICU admission, this immunosuppression state contributing to the occurrence of secondary bacterial infections [55]. In line with previous studies, our data indicated that leukocytosis and ICU admission are independent risk factors that are associated with bacterial infections [11]. Interestingly, the presence of these co-infections was not identified as a mortality predictor in the current study. However, the literature data are contrasting in this regard, with reports indicating escalating mortality rates in patients with infectious comorbidities during the SARS-CoV-2 pandemic, while other studies failed to demonstrate this mutual link [11,56,57]. However, Shafran et al. report that secondary bacterial infections may have a notable impact on patients’ prognosis, with a 2.7-fold increase in the risk of death in COVID-19 patients [12]. Designing predictive models for the risk stratification of co-infection or bacterial super-infection in patients with COVID-19 could be helpful for the optimal use of diagnostic tests, as well as for determining the need for antibiotic treatment. Gianella et al. created a validated, easy-to-use prediction model that allows risk stratification of bacterial co-infection (low, intermediate, and high), based on leukocyte count, procalcitonin, and Charlson comorbidity index values [58].

CRP and length of hospital stay are intrinsic risk factors associated with mortality that were identified by multivariate logistic regression. CRP has been previously identified as a relevant mortality predictor in several studies, including research conducted in the same geographical area [52,59]. The possible evolution of patients towards a hyperinflammatory status and cytokine storm during the course of COVID-19 pneumonia, with devastating tissue effects, has led to the extensive use of drugs with a role in modulating the immune response [60], as the administration of glucocorticoids significantly reduces the risk of mortality in patients with a severe form of COVID-19 [61]. Moreover, the interleukin-1β receptor antagonist anakinra has been proven to be safe and effective in reducing in-hospital mortality in patients with moderate and severe forms of COVID-19, especially in those with a hyperinflammatory syndrome, and a CPR value above 100 mg/L [62]. The use of immunomodulators is, however, accompanied by the following dilemma: could these drugs increase the rate of bacterial superinfections in patients with COVID-19? A study by Calverley indicates a significant increase in the risk of bacterial pneumonia in patients with a chronic respiratory disease who have been treated with glucocorticoids [63]. Buckley et al. refute the immunosuppressive role of the IL-1β receptor blockade, suggesting that its use is not associated with an increased risk of infection. The effectiveness of monoclonal antibodies is doubled by an adequate safety profile, a recent meta-analysis reporting a decrease of the mortality risk of patients with COVID-19, without increasing the risk of secondary bacterial infections [64]. Conversely, by attenuating the inflammatory response, the use of anakinra may lead to a delay in the diagnosis of infection, thus increasing the number of fatal infections [65]. The safety of IL-1β receptor antagonist administration in patients with severe COVID-19 and confirmed bacterial superinfections needs to be demonstrated in clinical trials, as the literature data that indicate its good safety profile are currently limited to case series [66].

Knowledge of the predictive factors for the need for antibiotic treatment can improve not only the management of antibiotherapy, but also the general prognosis of the patient. A meta-analysis has identified higher antibiotic prescription rates amongst elderly and critically ill patients who required mechanical ventilation [6]. In the study we carried out, cardiovascular comorbidities and the consolidation aspect of CT play a central role in predicting the need for antibiotic therapy. The bidirectional relationship between cardiovascular disease and COVID-19 is well-known [67,68]. As already discussed, pre-existing cardiovascular comorbidities are an independent risk factor for adverse events in patients with COVID-19, and the presence of infection is associated with an increased incidence of cardiovascular complications [69,70,71]. Thus, the validation of cardiovascular diseases in predicting the need for antibiotic treatment in this study is not surprising, considering their polymorphic pathophysiological mechanisms and the associated high-risk profile.

Pulmonary consolidations detected by CT scans are characteristic of bacterial pneumonia. In particular, their presence is also observed in patients with COVID-19, with a higher prevalence compared to non-COVID viral pneumonia [72,73]. The difficulty of differential diagnosis between COVID-19 pneumonia per se and the co-existence of an associated bacterial lung infection in the context of lung consolidations on CT was the determining factor for the antibiotic prescription by clinicians and represented a predictive factor for the need for antibiotic treatment in admitted patients.

To summarize, to avoid antibiotic therapy misuse, precise tools for differential diagnosis between viral and bacterial infection are increasingly required. Furthermore, encouraging clinicians to adhere to specific guidelines and to implement regionally tailored, cost-effective policies may facilitate appropriate antibiotic use in patients with viral respiratory infections.

### Limitations of the Study

First, the inclusion of a relatively limited number of patients led to a rather modest rate of diagnosis of bacterial infections. Consequently, we did not perform an analysis of the antibiotic resistance profiles in the identified microbial strains. Secondly, the assessment of procalcitonin levels was limited, as only a semi-quantitative assay was available in our hospital laboratory. Finally, the information regarding the COVID-19 vaccination status of the patients was not included in the statistical analysis due to the heterogeneity of the data. The rather low vaccination rate recorded in the region where the study was conducted (~37% of the eligible population), the existence of three vaccines available on the Romanian market and the identification of a large number of patients with an incomplete vaccination schedule could have caused bias in the analysis and substantially influenced the reported results.

## 4. Materials and Methods

### 4.1. Study Design and Patient Characteristics

The medical files of patients hospitalized during the fourth Romanian pandemic wave, between the 1 September and 30 November 2021 at the Clinical Hospital for Infectious Diseases “Saint Parascheva” (Iasi, Romania), were analyzed retrospectively and the data were extracted by two investigators. We conducted a unicentric retrospective observational study that included admitted patients with moderate, severe, and critical forms of COVID-19.

The inclusion criteria were as follows: patients with an age over 18 years and a confirmed diagnosis of SARS-CoV-2 infection by reverse transcription polymerase chain reaction. The microbiological tests (blood culture, culture of respiratory secretions and urine culture) were performed at admission or during the patients’ hospital stay. We excluded patients with severe or terminal pathologies, such as dialyzed chronic kidney disease, advanced heart failure, end-stage liver disease, active autoimmune diseases, immunosuppressive therapy, or malignancies. We also excluded patients who required early or emergent transfer to another service due to non-infectious comorbidities, patients transferred from other services and patients who recently benefited from in-hospital or ambulatory medical care (less than 14 days before admission), whether or not they had undergone invasive explorations.

After checking the inclusion and exclusion criteria, a total of 280 subjects were selected and further divided into 2 study groups. The first group consisted of 227 patients who received antibiotic regimens, while the control group included 53 patients with similar demographic and clinical characteristics, but without antibiotic treatment during their hospital stay.

### 4.2. Data Collection

After reviewing the medical files, the records were converted into a computerized database, entirely removing the identification data. Baseline demographic and clinical parameters (temperature and oxygen saturation), medical history, laboratory analyses, imaging results, and evolution during hospitalization were recorded. The laboratory tests included the patients’ complete blood count, common biochemical profile (kidney and liver functions, ferritin and fasting blood glucose), D-dimers and inflammatory biomarkers, pro-calcitonin (semi-quantitative dosage), C-reactive protein (CRP), erythrocyte sedimentation rate (ESH) and fibrinogen. Bacterial co-infections or secondary infections were confirmed by culture.

Microbiological samples were collected following the independent indication of the attending physician, by taking into account certain clinical and biological parameters. The exact extension and severity of the pulmonary lesions were assessed via computed tomography (CT), while specific microbiological determinations were performed according to hospital laboratory protocols. For testing the antibiotic sensitivity, either the disk diffusion method or the Microscan automatic antibiogram was used, which is capable of both identifying the microorganism and determining its resistance to antibiotics. Interpretation of the inhibition zone diameters was performed using the EUCAST table, with the breakpoints in effect at the time of strain isolation [74].

Antibiotic treatment was at the discretion of the attending physician, taking into consideration the clinical characteristics that are suggestive of a bacterial infection (fever, mucopurulent expectoration and presence of pulmonary rales) and the high levels of inflammatory biomarkers (procalcitonin, CRP, ESR and fibrinogen), as well as the aspect and the extension of pulmonary lesions (presence of pulmonary consolidations). The recommendation of the WHO not to initiate antibiotic treatment in patients with a moderate form of COVID-19 who do not exhibit elements that are suggestive of bacterial infection was accordingly respected. The WHO guidelines also recommend initiating antibiotic treatment in patients with a severe form of COVID-19 to cover the spectrum for all the possible pathogens. Antibiotic use before admission was carefully documented. For the in-hospital regimens, we mentioned the class of antibiotics, treatment duration, number of antibiotics per patient, and antibiotic treatment adjustment. The current WHO guideline recommendations concerning the opportunity to de-escalate empiric antibiotic treatment were followed [75]. In addition, we recorded the use of antivirals (e.g., favipiravir and remdesivir) and monoclonal antibodies, such as the IL-6 receptor blocker (tocilizumab) or inteleukin-1 receptor antagonist (anakinra). All the patients received anticoagulant therapy, according to the bleeding risk, while oxygen therapy was adjusted according to peripheral oxygen saturation.

Patients with refractory hypoxemia or severe dyspnea, who required non-invasive ventilation or mechanical ventilation, were transferred to the hospital’s intensive care unit (ICU), being further classified as patients with critical forms of COVID-19. The moderate forms of the disease were defined by the presence of clinical or imaging aspects that were suggestive of COVID-19 pneumonia, with a peripheral oxygen saturation of ≥94%. The severe forms of pneumonia were defined as polypnea (>30 breaths/minute), peripheral oxygen saturation of ≤93% in room air, or the presence of infiltrates that affected more than 50% of the lung area [76]. Fever was defined as an axillary temperature higher than 37.5 degrees Celsius. Bacteremia was ascertained by the presence of positive blood cultures [77], while a positive urine culture consisted of >100,000 colony-forming units/mL [78]. Bacterial infection was defined either as a co-infection, an infection confirmed from a microbiological test performed at initial presentation, or a secondary infection confirmed during the hospital stay [6]. The initiation of an antibiotic regimen within the first 24 h of admission designated the empirical approach, while an adjusted antibiotic treatment was defined as changing the initially administered antibiotic [79].

### 4.3. Ethics

The study was conducted in conformity with the principles contained in the Declaration of Helsinki and was accepted by the Ethics Committee of the Clinical Hospital of Infectious Diseases Saint Parascheva, Iași (approval number: 30, date of approval: 27 September 2022). Given the retrospective nature of the study, the hospital’s standard informed consent was provided by every patient at admission.

### 4.4. Statistical Analysis

We used the Kolmogorov–Smirnov test to evaluate the normal distribution of the continuous variables in the included patients. Abnormally distributed continuous variables were expressed as medians with interquartile ranges (IQRs), while normally distributed variables were expressed as means ± standard deviations (SDs). Categorical variables were depicted as absolute numbers and percentages. The *t*-test and Mann–Whitney U test were used for the comparative analysis between the two groups. To assess the equal variances for the categorical variables, we performed Levene’s test. The risk factors associated with bacterial infection and the predictors of a poor prognosis were identified via multivariate logistic regression and a Hosmer–Lemeshow preliminary test indicated the goodness-of-fit for the logistic regression model. The diagnostic accuracy of the inflammatory biomarkers was evaluated by receiver operating characteristic (ROC) analysis, with the subsequent comparison of the areas under the curve (AUC). The cut-off values for maximal sensitivity and specificity were determined using the Youden index. In all situations, the threshold of statistical significance was set at 0.05. The software used for conducting the statistical analysis was SPSS Statistics, version 23.0 (IBM, Armonk, NY, USA).

## 5. Conclusions

In conclusion, this study highlights that antibiotic treatment is routinely administered in patients hospitalized for COVID-19, often without being directly related to the presence of bacterial infection. The initial risk stratification using certain parameters associated with a poor prognosis is of utmost importance, as it may allow timely therapeutic interventions to reduce mortality, and it may also improve the quality of antimicrobial stewardship in COVID-19 patients.

## Figures and Tables

**Table 1:** Baseline characteristics and comorbidities.

Parameter	Total (*n* = 280)	Antibiotic Treatment (*n* = 227)	Without Antibiotics (*n* = 53)	*p*-Value
**Age**	**60.4 (15.2)**	**60.7 (15.1)**	**59.9 (15.8)**	0.273
<29	12 (4%)	8 (3.5%)	4 (7.5%)	
30–39	22 (8%)	21 (9.3%)	1 (1.9%)	
40–49	28 (10%)	16 (7%)	12 (22.6%)	
50–59	54 (19%)	51 (22.5%)	3 (5.7%)	
60–69	76 (27%)	60 (26.3%)	16 (30.2%)	
70–79	57 (21%)	42 (18.5%)	15 (28.3%)	
>80	31 (11%)	29 (12.8%)	2 (3.8%)	
**Gender**				
Female	142 (51%)	112 (49.3%)	30 (56.6%)	0.483
Male	138 (49%)	115 (50.7%)	23 (43.4%)	
**Disease severity**				
Moderate	146 (52%)	125 (55.1%)	21 (39.6%)	0.324
Severe	98 (35%)	69 (30.4%)	29 (54.7%)	
Critical	36 (13%)	33 (14.5%)	3 (5.7%)	
**Temperature °C** **> 37.5 °C**	119 (42%)	97 (42.7%)	22 (41.5%)	0.760
**Peripheral oxygen saturation < 93%**	125 (44.6%)	103 (44.9%)	22 (41.5%)	0.456
**Length of stay in hospital (days)**	10 (10–11)	11 (10–11)	9 (6–9)	0.032
**Comorbidities**				
Cerebrovascular diseases	36 (12.8%)	30 (13.2%)	6 (11.3%)	0.419
Cardiovascular diseases	135 (48.2%)	129 (56.8%)	6 (11.3%)	**<0.01**
Chronic digestive diseases	46 (16%)	36 (15.9%)	10 (18.9%)	0.594
Chronic renal disease	17 (6%)	14 (6.2%)	3 (5.7%)	0.278
Diabetes	62 (22.1%)	58 (25.6%)	4 (7.5%)	0.750
Obesity	68 (24%)	60 (26.4%)	8 (15.1%)	**<0.01**

Data are expressed as absolute numbers and percentages (%), median and interquartile range (IQR 25–75), or mean ± standard deviation (SD).

**Table 2:** Laboratory and imagistic findings.

	Total (*n* = 280)	Antibiotic Treatment (*n* = 227)	Without Antibiotics (*n* = 53)	*p*-Value
**Laboratory findings**				
White blood cell count, ×10^9^/L <4 4–10 >10	48 (17.1%) 158 (56.4%) 73 (26%)	42 (18.5%) 117 (51.5%) 68 (30%)	7 (13.2%) 41 (77.4%) 5 (9.4%)	0.610
Neutrophil to lymphocyte ratio %	4.4 (3.9–5.3)	5 (4.2–5.8)	3.1 (2.3–3.9)	0.470
C-reactive protein, mg/L >5	260 (92.85%)	215 (94.7%)	45 (84.9%)	**<0.01**
Fibrinogen, g/L	4.7 (4.5–4.9)	4.8 (4.6–4.9)	4.1 (3.4–5.1)	**0.01**
ESR, mm/h	62.4 (33.4)	62.4 (32.2)	62.2 (38.3)	0.608
D-dimer > 0.5 mg/L	136 (48.57%)	120 (52.9%)	16 (30.2%)	0.499
Ferritin > 350 ng/mL	163 (58.21%)	155 (68.3%)	28 (52.8%)	0.284
Procalcitonin <0.5 ng/mL 0.5–2 ng/mL 2–10 ng/mL	200 (71.4%) 65 (23.2%) 15 (5.3%)	154 (67.8%) 61 (26.9%) 12 (5.3%)	46 (86.8%) 4 (7.5%) 3 (5.7%)	**0.005**
Lactate dehydrogenase > 430 U/L	56 (20%)	53 (22.9%)	3 (5.7%)	0.638
Aspartate aminotransferase > 37 U/L	161 (57.5%)	137 (60.4%)	24 (45.3%)	0.140
Alanine aminotransferase > 40 U/L	112 (40%)	92 (40.5%)	20 (37.7%)	0.088
Serum creatinine, mg/dL > 1.1	86 (31%)	76 (33.5%)	10 (4.4%)	0.341
Serum urea > 50 mg/dL	98 (35%)	84 (37%)	14 (26.4%)	0.254
**Imagistic findings**				
Ground-glass opacities	175 (62.5%)	156 (55.7%)	19 (35.8%)	**0.022**
Pulmonary consolidation	36 (12.9%)	34 (15%)	2 (3.8%)	**0.034**

Data are expressed as absolute numbers and percentages (%), median and interquartile range (IQR 25–75), or mean ± standard deviation (SD). ESR—erythrocyte sedimentation rate.

**Table 3:** Associated therapeutic regimens.

	Total (*n* = 280)	Antibiotic Treatment (*n* = 227)	Without Antibiotics (*n* = 53)	*p*-Value
**Antiviral agents** Remdesivir Favipiravir	59 (21%) 96 (34.2%)	59 (26%) 76 (33.5%)	0 (0%) 20 (37.7%)	0.042
**Monoclonal antibodies** Anakinra Tocilizumab	40 (14.2%) 48 (17.1%)	38 (16.7%) 43 (18.9%)	2 (3.8%) 5 (9.4%)	0.108 0.125
Glucocorticoids	170 (61%)	136 (59.9%)	34 (64.1%)	0.091

Data are expressed as absolute numbers and percentages (%).

**Table 4:** Evolution.

**Admission to ICU**	41 (14.6%)	38 (16.7%)	3 (5.7%)	**0.07**
**Death**	31 (11%)	27 (11.9%)	6 (11.3%)	**0.01**

Data are expressed as absolute numbers and percentages (%).

**Table 5:** Correlations between the need for antibiotic treatment and certain clinical and biological parameters.

Parameter	Antibiotic Treatment	
	R	*p*-Value
O_2_ saturation at admission	−0.165	0.006
Cardiovascular comorbidities	0.340	<0.001
Diabetes mellitus	0.170	0.004
Obesity	0.104	0.084
Pulmonary consolidation via CT	0.427	<0.001
Age	0.033	0.579
Leukocytosis	0.148	0.013
Fibrinogen	0.119	0.046
CRP	0.216	<0.001
Ferritin	0.170	0.004
Aspartate aminotransferase	0.121	0.044
Serum creatinine	0.073	0.202
Lactate dehydrogenase	0.177	0.003
D-Dimers	−0.019	0.747
Fasting blood glucose	0.195	0.001

**Table 6:** A multivariable model that predicts the need for antibiotic treatment.

Model Summary					
Model	R	R Square	Adjusted R Square	Std. Error of the Estimate	*p*-Value
1	0.427 ^a^	0.182	0.179	0.356	<0.01
2	0.505 ^b^	0.255	0.250	0.340	<0.01

a. Predictors: pulmonary consolidation on CT (constant). b. Predictors: pulmonary consolidation on CT; cardiovascular comorbidities (constant).

**Table 7:** Multivariate logistic regression analysis of bacterial infection risk factors.

Parameter	B	S.E.	Wald	*p*-Value	Exp (B)	95% Confidence Interval	
						Lower	Upper
**ICU admission**	1.538	0.497	9.570	0.002	4.657	1.757	12.342
**Leukocytosis**	0.001	0.001	8.404	0.004	1.00014	1.000046	1.00024

**Table 8:** Multivariable logistic regression analysis of mortality risk factors.

Parameter	B	S.E.	Wald	P	Exp (B)	95% Confidence Interval	
						Lower	Upper
**Length of hospital stay**	0.375	5.657	14.090	0.001	1.456	0.266	2.730
**C-reactive protein**	0.017	0.365	5.216	0.028	1.017	0.009	0.133

**Table 9:** AUC analysis: the biomarkers’ capacity in predicting mortality.

Area under the Curve					
Test Result Variable(s)	Area	Std. Error	*p*-Value	Asymptotic 95% Confidence Interval	
				Lower Bound	Upper Bound
ESR	0.781	0.044	<0.0001	0.695	0.867
CRP	0.797	0.045	<0.0001	0.709	0.885
Fibrinogen	0.664	0.060	0.006	0.546	0.782

**Table 10:** Cut-off values for ESR and CRP.

Criterion	ESR Cut-Off (mm/h)	Se	Sp	CRP Cut-Off mg/L	Se	Sp
Youden’s index (maximum Se + Sp)	47.5	74.1%	56%	97.93	55.6%	34.5%
High-risk profile	51.5	74.1%	56%	67.01	74.1%	53.5%

ESR—erythrocyte sedimentation rate, CRP—C-reactive protein, Se = sensitivity; Sp = specificity.

## Data Availability

All necessary data are found within the text of the manuscript.
